# Supplementary material for: scLENS: data-driven signal detection for unbiased scRNA-seq data analysis
Source: Nat Commun. 2024 Apr 27;15:3575. doi: 10.1038/s41467-024-47884-3 (PMC11519519; doi:10.1038/s41467-024-47884-3)
Supplement: Supplementary file 1 — Supplementary information [file 41467_2024_47884_MOESM1_ESM.pdf]

Supplementary Information

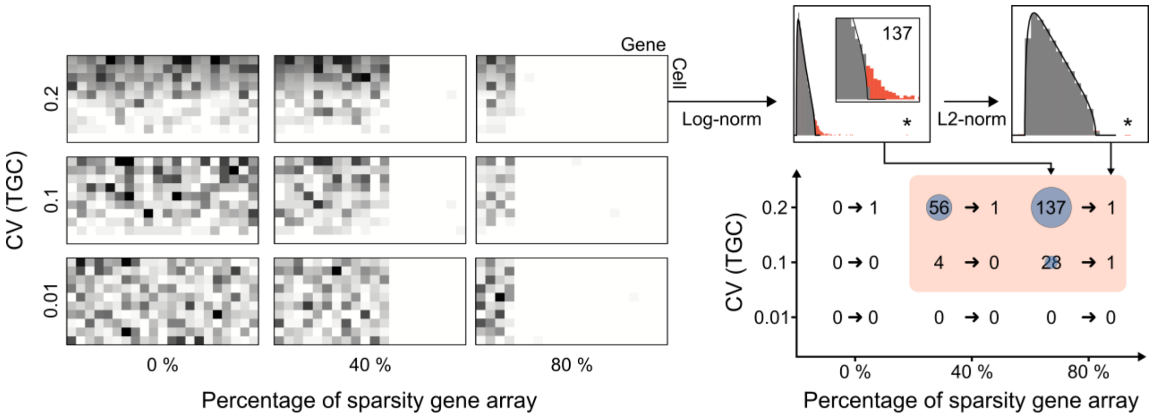

Supplementary Figure 1. Effect of log and L2 normalizations on signal detection in random matrices with varying gene sparsity and TGC variance. Nine random matrices were constructed, each with varying levels of sparse gene percentages (0%, 40%, and 80%) and coefficients of variation (CVs) in TGCs of 0.01, 0.1, and 0.2. Applying log normalization to sparse data with a high CV of TGC results in the excessive detection of false signals within the random matrix, as indicated by the numbers to the left of the arrow in the orange box. These distortions are effectively removed by additional L2 normalization, as seen in the numbers to the right of the arrow in the orange box.

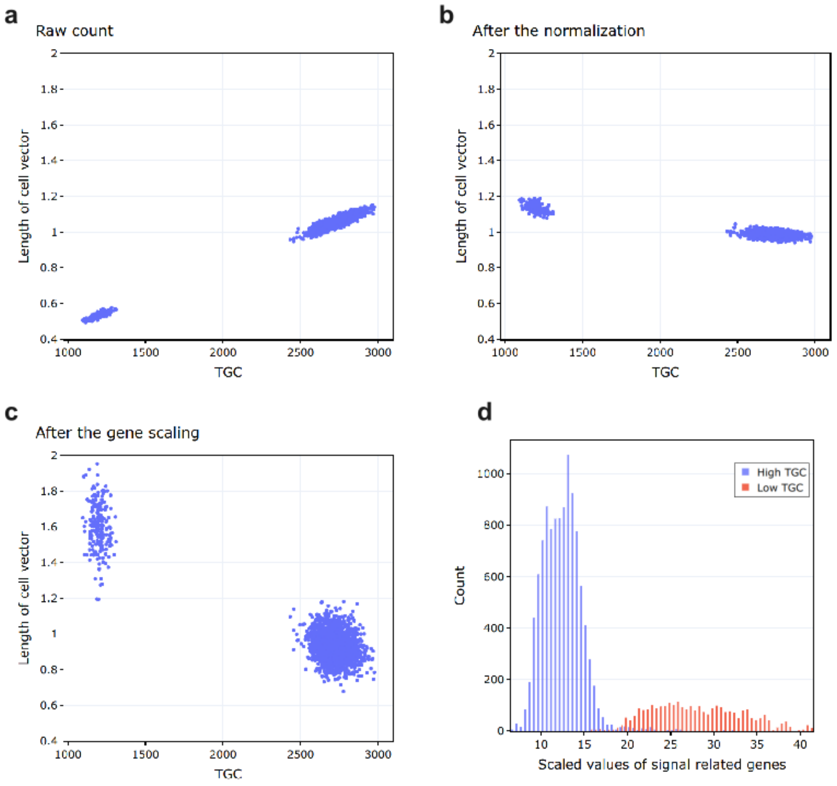

Supplementary Figure 2. Change in lengths of cell vectors during normalization of the random matrix. a, Because the random matrix (Fig. 2k) consists of 1,600 cell vectors with high TGC and 400 cell vectors with low TGC, the length of cell vectors with high TCG is longer than the length of cell vectors with low TGC. **b**, The lengths of cell vectors with low and high TGC become similar after normalization, the first step of log normalization. **c**, However, the lengths of cell vectors with low TGC excessively lengthen after gene scaling, the last step of log normalization. This reintroduces variance in cell vector lengths that were removed by normalization. **d**, To identify the source of variance reintroduction in cell vector lengths, the distribution of elements in cell vectors with low TGC (c) was compared to that in cell vectors with high TGC (c) after gene scaling. While two distributions of elements from dense gene arrays are similar, the distribution of elements in sparse genes of cells with low TGC dominates that of cells with high TGC. This means that the exaggerated length of cell vectors with low TGC during log normalization is due to the over-amplification of non-zero values in sparse genes of cell vectors with low TGC (red histogram).

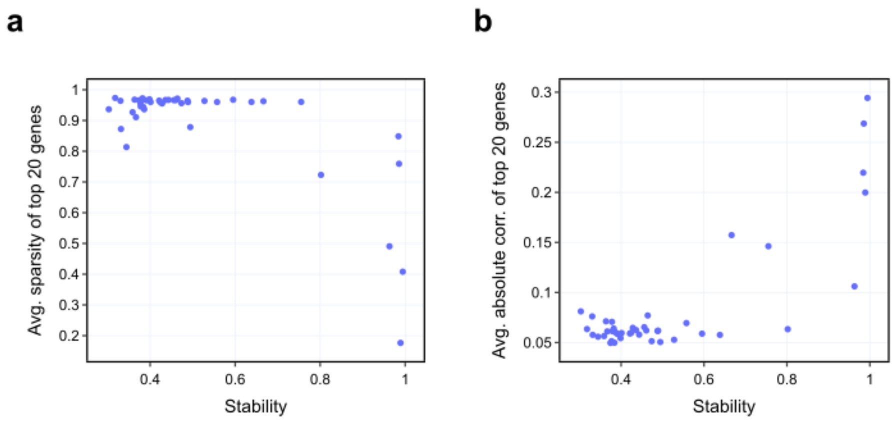

Supplementary Figure 3. The top 20 genes contributing to each low stability signal are sparse and exhibit low correlations with each other. a-b, Using RMT-based noise filtering, 42 signals were detected from ZhengMix data (Fig. 3h). Their respective stabilities were then obtained using signal robustness test. To discern the gene properties contributing to each signal, the top 20 genes most correlated to each signal were obtained by calculating correlations between normalized gene vectors and signal vectors, as measured by the dot product. Then, these genes' mean sparsity levels (a) and the average of their correlations were calculated (b). As the stability of a signal decreases, the average sparsity level of genes contributing to this signal increases (a), and their average correlation decreases (b). Therefore, the low-quality signals were likely to be raised due to spurious correlations between genes with high sparsity levels caused by dropout.

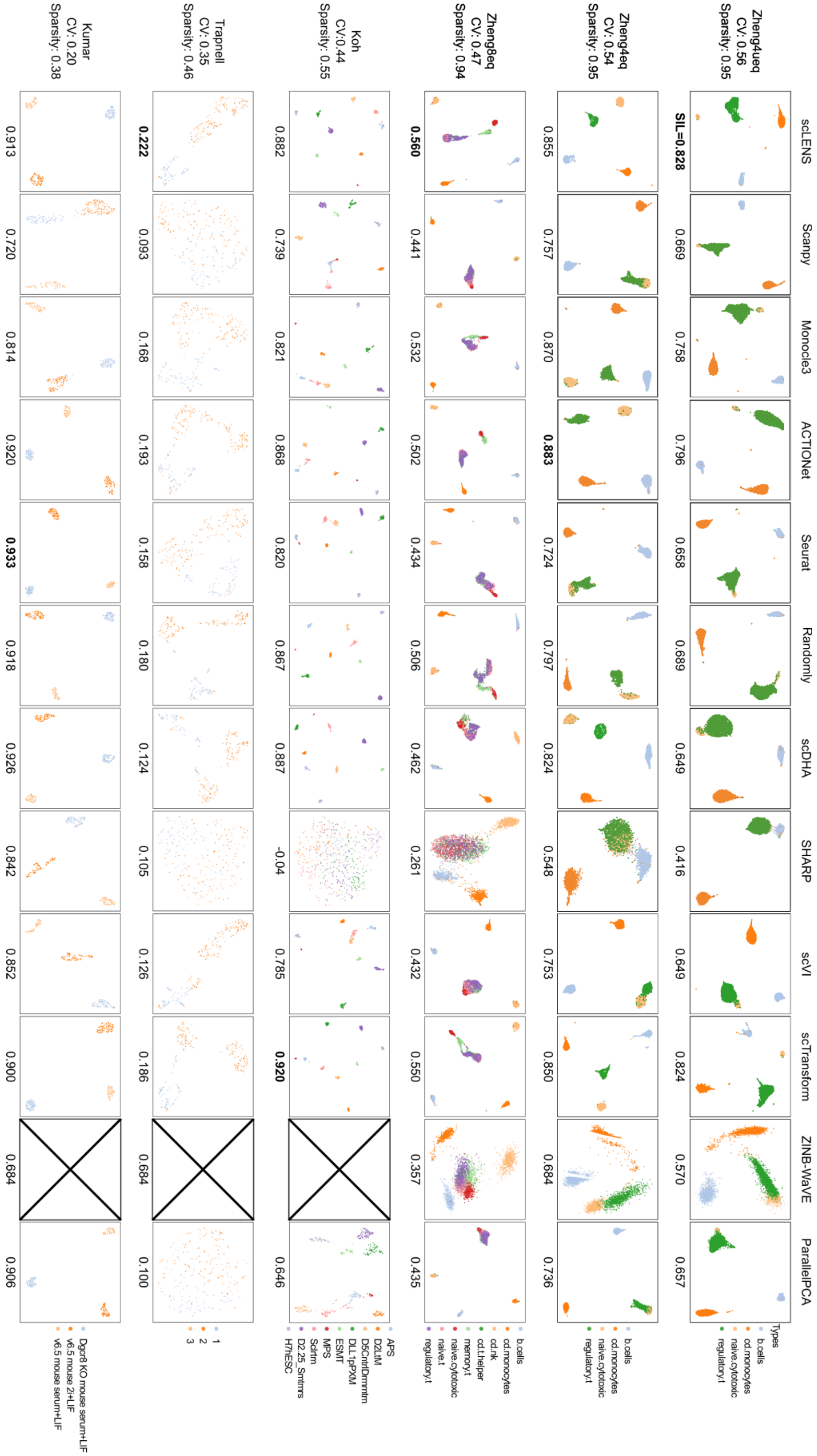

Supplementary Figure 4. Comparison of 2D UMAP embeddings obtained from 12 packages, including scLENS. All 2D embeddings were obtained using UMAP with a minimum distance of 0.01 and 15 nearest

neighbors. Cells were colored by their ground truth labels. Note that the UMAP embeddings of ZINB-WaVE for the Koh, Trapnell, and Kumar datasets are unavailable due to computational errors.

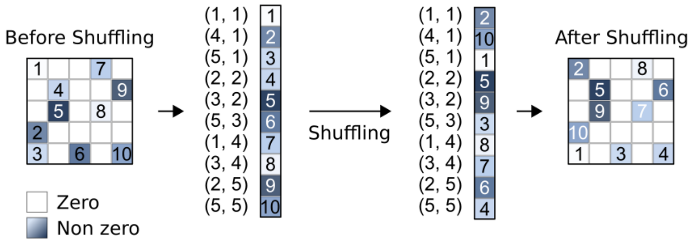

Supplementary Figure 5. Schematic figure for non-zero value shuffling. To eliminate non-binary information from data (Fig. 5), the non-zero values are extracted as an array, and its elements are shuffled. The shuffled values then replace the original values.

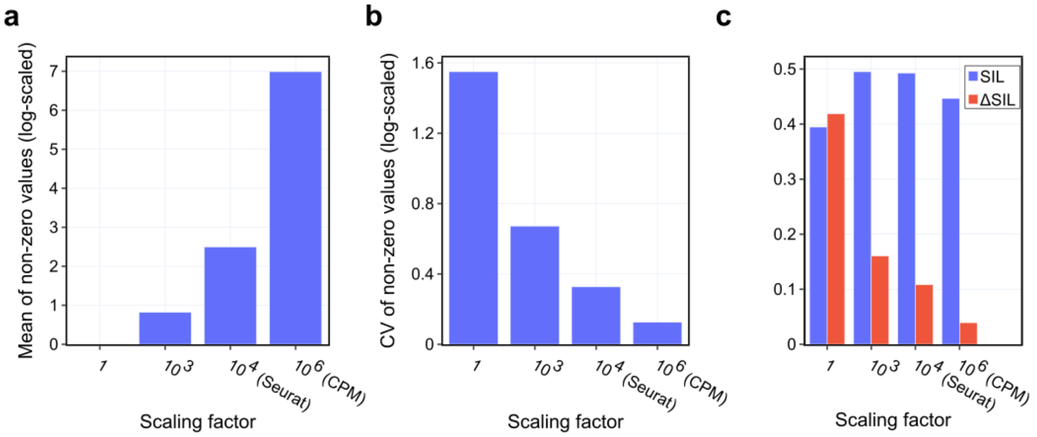

Supplementary Figure 6. Impact of large scaling factor on data binarization in ZhengMix data. a, After log transformation, multiplying non-zero values by a scaling factor larger than 1 results in a greater gap between zero and non-zero values. **b,** The coefficient of variation (CV) in these increased non-zero values decreases after applying a log transformation as the scaling factor increases. **c**, The amplification gap between zero and non-zero values (a), coupled with the reduced CV in non-zero values (b) due to the large scaling factor, leads to a decrease in data ΔSIL, indicating data binarization. Source data are provided as a Source Data file.

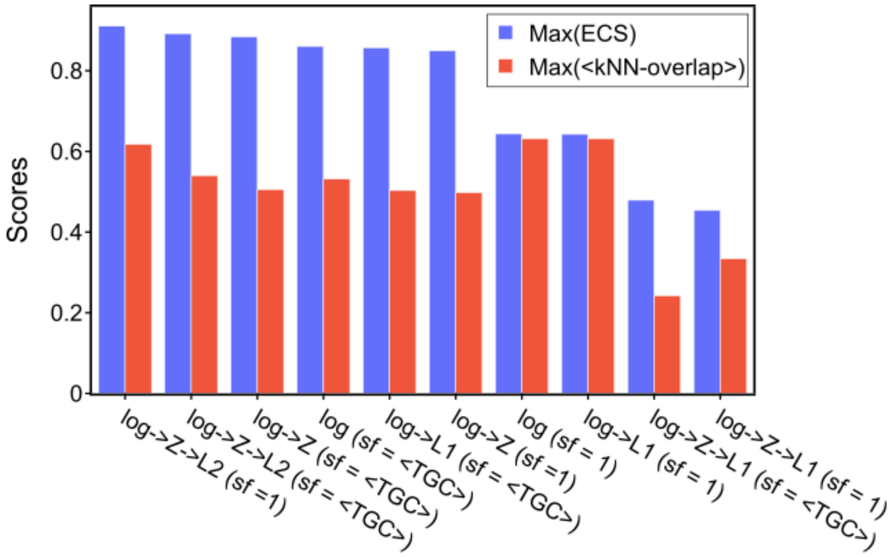

Supplementary Figure 7. Performance comparison of ten different transformations. The performance evaluation was conducted using ECS and the average kNN-overlap scores at the optimal number of PCs on five deeply sequenced datasets utilized in the Ahlmann-Eltze study and 19 datasets with ground truth labels used in the clustering benchmark (Supplementary Table 2). In terms of the ECS score (blue bar), which indicates the clustering performance, L2 normalization following log normalization with a scaling factor of 1 (log -> Z -> L2 (sf = 1)) showed the best performance. In contrast, L1 normalization following log normalization (log -> Z -> L1 (sf = 1)) exhibited the lowest. In terms of the average kNN-overlap score (red bar), log normalization without gene scaling using a scaling factor of 1 (log (sf = 1)) demonstrated the best performance. However, its clustering performance is sub-optimal. On the other hand, L2 normalization following log normalization with a scaling factor of 1 (log -> Z -> L2 (sf = 1)) exhibited competitive performance in terms of the average kNN-overlap score. Furthermore, the transformation performance using the average TGC for a scaling factor (log -> Z -> L2 (sf = <TGC>)) instead of a scaling factor of 1 was downgraded due to the Information loss caused by data binarization (Supplementary Fig. 6). Source data are provided as a Source Data file.

	Pre-processing					Dimensionality reduction	# Signals (Defaults)	Version
scLENS	Library size normalization	log transformation		gene scaling	L2 norm.	Robust signal selection (RMT-based filtering) post-noise filtering	auto	-
Seurat	Library size normalization	log transformation	feature selection (HVG)	gene scaling		PCA(irlba)	50	5.0.0
Scanpy	Library size normalization	log transformation	feature selection (HVG)	gene scaling		Standard PCA	50	1.9.3
Monocle3	Library size normalization	log transformation		gene scaling		PCA(irlba)	50	1.3.1
Randomly	Library size normalization	log transformation		gene scaling		Denoising with RMT-based filtering + Feature selection based on SNR	auto	0.1.0
scVI	Library size normalization	log transformation	feature selection (HVG)			Neural Network	10	1.0.4
ACTIONet	Library size normalization	log transformation				Matrix Factorization	50	3.0.2
scDHA		log transformation	feature selection (kernel autoencoder)			Stacked Bayesian Self-learning Network	20	1.2.1
SHARP		log transformation				Random projection	auto	1.1.0
ParallelPCA	Library size normalization	log transformation	feature selection (HVG)	gene scaling		Automatic PC selection based on the statistical significant of PCs	auto	2.12.0
ZINB-WaVE	Fit to the ZINB model					ZINB-WaVE procedure	2	1.22.0
scTransform	Pearson residual with gamma-Poisson distribution					PCA (irlba)	50	Seurat(5.0.0)

Supplementary Table 1. Dimensionality reduction (DR) workflows of the benchmarking methods

Data set	Data type	# of data	Protocol	Descripion	Ref.
Kho	Real read count	1	SMARTer	FACS purified H7 human embryonic stem cells	[48]
Kumar	Real read count	1	SMARTer	Mouse embryonic stem cells, cultured with different inhibition factors	[49]
Trepnell	Real read count	1	SMARTer	Human skeletal muscle myoblast cells, differention induced by low-serum medium	[50]
Zhengmix4eq	Real UMI	1	10x	Mixture of purified peripheral blood mononuclear cells	[51]
Zhengmix4ueq	Real UMI	1	10x	Mixture of purified peripheral blood mononuclear cells	[51]
Zhengmix8eq	Real UMI	1	10x	Mixture of purified peripheral blood mononuclear cells	[51]
ZhengMix	Real UMI	10	10x	Mixture of purified peripheral blood mononuclear cells	[51]
Sim. Tabula muris	Simulated UMI	10	scDesign2	Data generated by training 10 cell types of Tabula muris data	[44, 47]
Sim. T cell	Simulated UMI	13	scDesign2	Data generated by training by 13 cell types of T-cells from Cross-tissue Immune Cell Atals	[6, 44]
E-MTAB-10148	Real UMI	2	SmartSeq3	Primary mouse fibroblasts derived from the tail of a male adult mouse, F1 offspring of a C57 x CAST cross.	[61]
E-MTAB-13293	Real UMI	1	Smartseq3xpress	Perinatal liver Hematopoietic Stem Cells (HSCs) in Mus musculus	[62]
E-MTAB-8735	Real UMI	2	SmartSeq3	Fibroblast and HEK cells from mixed sample (Mus musculus, Homo sapiens)	[58]
SmartSeq3 siRNA_knockdown	Real UMI	1	SmartSeq3	Fibroblast siRNA Knockdown Data	[61]
mcSCRBseq	Real UMI	1	mcSCRB-seq	JM827 mouse embryonic stem cells	[57]
E-MTAB-11467	Real UMI	8	Smartseq3xpress	HEK293FT cells, K562 cells, and human peripheral blood mononuclear cells	[60]
Darmanis	Real read count	1	SMARTer	Human brain cells	[54]
Deng	Real read count	1	Smart-Seq2	Cells from mouse zygotes to late blastocyst stages, and adult liver cells, using crosses between CAST/Ei and C57BL/6 mouse strains	[53]
Goolam	Real read count	1	Smart-Seq2	Cells from mouse embryos at various stages (2-, 4-, 8-, 16-, and 32-cell)	[55]
Li	Real read count	1	SMARTer	Cells from colorectal tumors and their microenvironments	[56]

Supplementary Table 2. Real and simulation datasets for benchmarking. It includes 58 datasets: 39 datasets (16 real and 23 simulated) with ground truth labels, independently obtained from scRNA-seq analysis tools, and 19 deeply sequenced datasets without ground truth, comprising 15 UMI count datasets and 4 read count datasets.

Manual for the code

scLENS: Data-driven signal detection for unbiased scRNA-seq data analysis

Table of Contents

1. Installation of Julia Language
   - Windows
   - Linux (Debian)
2. Download scLENS from GitHub
3. Install Required Packages
4. Convert 10x Files
5. Run scLENS

Installation of Julia Language

Warning about Dependency Packages: To ensure optimal performance and compatibility, it's recommended to use the latest versions of dependent packages. Potential compatibility issues may arise if outdated versions are used.

Windows

1. **Download Julia**: Visit the Julia Downloads page.
2. **Install Julia**: Run the downloaded .exe file.
3. **Environment Path**: Check the box to add Julia to your system PATH during installation.

Linux (Debian)

1. **Download Julia**: Use wget to download the pre-compiled binary. `bash wget https://julialang-s3.julialang.org/bin/linux/x64/1.6/julia-1.6.7-linux-x86_64.tar.gz`
2. **Install Julia**: Extract the downloaded archive. `bash tar zxvf julia-1.6.7-linux-x86_64.tar.gz`
3. **Environment Path**: Add Julia to your PATH. (Please change the name of home folder) `bash echo 'export PATH="$PATH:/home/users/julia-1.6.7/bin"' >> ~/.bashrc source ~/.bashrc` This will install Julia and make it accessible from the command line.

Download scLENS from GitHub

1. Navigate to the scLENS GitHub repository.
2. Click on the Code button and download the ZIP file or clone the repository using git: `bash git clone https://github.com/yourusername/sclens.git`

Install Required Packages

1. Navigate to the downloaded sclens folder.
2. Open a terminal in that folder and run: `bash julia installation.jl`

Convert 10x Files to a JLD File

To convert 10x files to a JLD file, you can use the `convert_to_jld.jl` script. The folder containing your 10x files should include the following three files:

- `matrix.mtx.gz`
- `barcodes.tsv.gz`
- `features.tsv.gz`

Run the following command to perform the conversion:

```
julia convert_to_jld.jl /path/to/10xfiles/folder/ -d data/ -n test.jld2
```

Note: -d specifies the directory where the output JLD file will be saved. -n specifies the name of the output JLD file.

The file extension must always be .jld2.

Run scLENS

To run scLENS, you can use the following options:

- `--true_label`, `-t`: Specify a CSV file containing true cell-type labels for each cell. The number of labels must match the number of rows in the input matrix.
  Type: String
  Default: None

- `--plot`, `-p`: Enable this option to save three types of plots to the output directory: eigenvalue distribution, UMAP embedding, and signal stability plot.
  Action: store_`true`

- `--device`: Select the computing device for scLENS. Options are 'gpu' or 'cpu'.
  Type: String
  Default: `gpu`

- `--out_dir`, `-d`: Specify the directory where output files will be saved.
  Type: String
  Default: `tempname()`

- `--scaling` : Choose the scaling method for gene-scaling. Options are "mean" for z-score scaling and "median" for median scaling.
  Type: String
  Default: `mean`

- `--out_type`, `-o`: Choose the format for the output files. Options are 'julia' (jld2), 'python' (npz), 'r' (RData), 'anndata' (h5ad), 'seurat' (rds), and 'csv'. Note that selecting 'csv' will only output PCA results.
  Type: String
  Default: `julia`

  Additional Details:

  1. **Seurat Dependency**: To use the saving options for 'seurat', seurat should be installed in your R environments respectively.
  2. **R Environment for Seurat**: The R_HOME environment variable for RCall.jl should point to the R home directory where seurat is installed. Refer to the RCall.jl installation guide for more details.

- arg1: Provide the path to the input file, which should be a CSV or JLD2 file. The file must contain a matrix with rows representing cells and columns representing genes.
  Required: true

Example usage:

```
julia scLENS.jl data/sim_Tcell_3.csv.gz --true_label data/sim_Tcell_3_l.csv --
out_dir out_dir --device cpu --out_type julia --plot
```

Output Details

After successfully running scLENS, a single output file will be generated in the directory specified by the `--out_dir` option. The format of this output file is determined by the `--out_type` option. This file will contain a dictionary with the following key-value pairs:

- `pca`: Reduced data after noise filtering based on Random Matrix Theory (RMT).
- `pca_n1`: Reduced data after both RMT noise filtering and signal stability tests.
- `sig_id`: Identifiers for robust signals.
- `L`: All eigenvalues.
- `L_mp`: Eigenvalues related to noise.
- `λ`: Tracy-Widom (TW) threshold value.
- `st_mat`: Signal stability matrix.
- `m_scores`: Mean stability vector.
- `sd_scores`: Standard deviation stability vector.
- `signal_evec`: Eigenvectors corresponding to signals.
- `signal_ev`: Eigenvalues corresponding to signals.
- `cell_id`: Identifiers for the input cells.
- `umap`: 2D UMAP coordinates.
- `umap_obj`: UMAP object including a UMAP graph (only included in JLD2 files).

Note: - Please utilize the reduced data with the key “`pca_n1`” for your analyses, rather than the data with the key “`pca`.” - If you wish to save the output as an RData file, make sure to install the R language on your system.

This single output file offers a comprehensive set of variables useful for analyzing and interpreting the results generated by scLENS.
