# Supplementary material for: scLENS: data-driven signal detection for unbiased scRNA-seq data analysis
Source: Nat Commun. 2024 Apr 27;15:3575. doi: 10.1038/s41467-024-47884-3 (PMC11519519; doi:10.1038/s41467-024-47884-3)
Supplement: Supplementary file 3 — Description of Additional Supplementary Files [file 41467_2024_47884_MOESM3_ESM.pdf]

Description of Additional Supplementary Files:

Supplementary Data 1: Data table comprising data properties, including the number of cells, level of sparsity, detected number of signals, and the goodness of fit of the eigenvalue distribution to the Marchenko-Pastur distribution.
