# Supplementary material for: scLENS: data-driven signal detection for unbiased scRNA-seq data analysis
Source: Nat Commun. 2024 Apr 27;15:3575. doi: 10.1038/s41467-024-47884-3 (PMC11519519; doi:10.1038/s41467-024-47884-3)
Supplement: Supplementary file 5 — Reporting Summary [file 41467_2024_47884_MOESM5_ESM.pdf]

nature portfolio

Corresponding author(s): Jae Kyoung Kim

Last updated by author(s): Mar 19, 2024

Reporting Summary

Nature Portfolio wishes to improve the reproducibility of the work that we publish. This form provides structure for consistency and transparency in reporting. For further information on Nature Portfolio policies, see our Editorial Policies and the Editorial Policy Checklist.

Statistics

For all statistical analyses, confirm that the following items are present in the figure legend, table legend, main text, or Methods section.

n/a	Confirmed	
☐	☒	The exact sample size (*n*) for each experimental group/condition, given as a discrete number and unit of measurement
☒	☐	A statement on whether measurements were taken from distinct samples or whether the same sample was measured repeatedly
☒	☐	The statistical test(s) used AND whether they are one- or two-sided<br>*Only common tests should be described solely by name; describe more complex techniques in the Methods section.*
☒	☐	A description of all covariates tested
☐	☒	A description of any assumptions or corrections, such as tests of normality and adjustment for multiple comparisons
☐	☒	A full description of the statistical parameters including central tendency (e.g. means) or other basic estimates (e.g. regression coefficient) AND variation (e.g. standard deviation) or associated estimates of uncertainty (e.g. confidence intervals)
☒	☐	For null hypothesis testing, the test statistic (e.g. *F*, *t*, *r*) with confidence intervals, effect sizes, degrees of freedom and *P* value noted<br>*Give P values as exact values whenever suitable.*
☒	☐	For Bayesian analysis, information on the choice of priors and Markov chain Monte Carlo settings
☒	☐	For hierarchical and complex designs, identification of the appropriate level for tests and full reporting of outcomes
☒	☐	Estimates of effect sizes (e.g. Cohen's *d*, Pearson's *r*), indicating how they were calculated

Our web collection on statistics for biologists contains articles on many of the points above.

Software and code

Policy information about availability of computer code

Data collection	No additional code were utilized for the data collection
Data analysis	* Packages and their version used for the benchmark studies<br>- Seurat (R) - Version 5.0.0<br>- Scanpy (Python) - Version 1.9.3<br>- Monocle3 (R) - Version 1.3.1<br>- Randomly (Python) - Version 0.1.0<br>- scVI (Python) - Version 1.0.4<br>- ACTIONet (R) - Version 3.0.2<br>- scDHA (R) - Version 1.2.1<br>- SHARP (R) - Version 1.1.0<br>- ParallelPCA (R) - Version 2.12.0<br>- ZINB-WaVE (R) - Version 1.22.0<br>- scTransform (R) - Version specified as Seurat(5.0.0)<br>- scDesign2 (R) - Version 1.0.0<br><br>R-based packages were run using R version 4.3.2.<br>Python-based packages were run using Python version 3.11.5

scLENS was built using Julia 1.8.5 and tested with Julia 1.10.0.
All dependent packages of scLENS and their versions are as follows:

ArgParse v1.1.4, CSV v0.10.12, CUDA v5.1.1, CairoMakie v0.11.4, Clustering v0.15.6, CodecLz4 v0.4.1, ColorSchemes v3.24.0, Colors v0.12.10, DataFrames v1.6.1, Distances v0.10.11, Glob v1.3.1, InlineStrings v1.4.0, JLD2 v0.4.41, Makie v0.20.3, Muon v0.1.1, NPZ v0.4.3, NaNStatistics v0.6.33, Pandas v1.6.1, ProgressMeter v1.9.0, RCall v0.13.18, StatsBase v0.33.21, Suppressor v0.2.6, UMAP v0.1.10, LinearAlgebra, Printf, Random, SparseArrays v1.10.0

The scLENS code and installation instructions are available at the GitHub link: https://github.com/Mathbiomed/scLENS.

For manuscripts utilizing custom algorithms or software that are central to the research but not yet described in published literature, software must be made available to editors and reviewers. We strongly encourage code deposition in a community repository (e.g. GitHub). See the Nature Portfolio guidelines for submitting code & software for further information.

Data

Policy information about availability of data

All manuscripts must include a data availability statement. This statement should provide the following information, where applicable:

- Accession codes, unique identifiers, or web links for publicly available datasets
- A description of any restrictions on data availability
- For clinical datasets or third party data, please ensure that the statement adheres to our policy

The real datasets used in this study are publicly available and can be accessed through the following sources: Koh, Kumar, and Trapnell datasets were obtained from the GitHub repository [https://github.com/markrobinsonuzh/scRNAseq_clustering_comparison]. Zheng datasets were obtained from 10x Genomics datasets [https://www.10xgenomics.com/resources/datasets]. The Tabula Muris dataset was obtained from the Tabula Muris Project [https://tabula-muris.ds.czbiohub.org/]. The immune cell dataset was obtained from the Cross-tissue Immune Cell Atlas [https://www.tissueimmunecellatlas.org]. The mouse fibroblasts dataset was obtained from ArrayExpress under the accession code E-MTAB-10148 [https://www.ebi.ac.uk/biostudies/arrayexpress/studies/E-MTAB-10148]. The perinatal mouse hematopoietic stem cells dataset was obtained from ArrayExpress under the accession code E-MTAB-13293 [https://www.ebi.ac.uk/biostudies/arrayexpress/studies/E-MTAB-13293]. The fibroblast and HEK cells dataset were obtained from ArrayExpress under the accession code E-MTAB-8735 [https://www.ebi.ac.uk/biostudies/arrayexpress/studies/E-MTAB-8735]. The siRNA KnockDown dataset was obtained from the GitHub repository [https://github.com/sandberg-lab/lncRNAs_bursting/tree/main/data]. The JM8 cells dataset was obtained from Gene Expression Omnibus (GEO) database under the accession code GSE103568 [https://www.ncbi.nlm.nih.gov/geo/query/acc.cgi?acc=GSE103568]. The HEK293FT, K562, and human PBMC cells datasets were obtained from ArrayExpress under the accession code E-MTAB-11467 [https://www.ebi.ac.uk/biostudies/arrayexpress/studies/E-MTAB-11467]. The human brain cells dataset was obtained from the Hemberg Lab repository [https://hemberg-lab.github.io/scRNA.seq.datasets/human/brain/#darmanis]. The scRNA-seq dataset with cells from mouse zygotes to blastocysts was obtained from the Hemberg Lab repository [https://hemberg-lab.github.io/scRNA.seq.datasets/mouse/edev/#deng]. The mouse embryos dataset was obtained from the Hemberg Lab repository [https://hemberg-lab.github.io/scRNA.seq.datasets/mouse/edev/#goolam]. The colorectal tumor cells dataset was obtained from the Hemberg Lab repository [https://hemberg-lab.github.io/scRNA.seq.datasets/human/tissues/#li]. Source data for Figs. 4-7 and Supplementary Figs. 6-7 have been provided with this paper. A selection of the real datasets and all simulated datasets used in this study are available in the GitHub repository [https://github.com/Mathbiomed/scLENS] and archived at Zenodo [https://doi.org/10.5281/zenodo.10839592].

Research involving human participants, their data, or biological material

Policy information about studies with human participants or human data. See also policy information about sex, gender (identity/presentation), and sexual orientation and race, ethnicity and racism.

Reporting on sex and gender	No applicable
Reporting on race, ethnicity, or other socially relevant groupings	No applicable
Population characteristics	No applicable
Recruitment	No applicable
Ethics oversight	No applicable

Note that full information on the approval of the study protocol must also be provided in the manuscript.

Field-specific reporting

Please select the one below that is the best fit for your research. If you are not sure, read the appropriate sections before making your selection.

[x] Life sciences   [ ] Behavioural & social sciences   [ ] Ecological, evolutionary & environmental sciences

For a reference copy of the document with all sections, see nature.com/documents/nr-reporting-summary-flat.pdf

Life sciences study design

All studies must disclose on these points even when the disclosure is negative.

Sample size: To minimize the sample size effect, we sampled ~3,000 cells from the generated ~60,000 simulated immune cells pool to create 13 simulated

	datasets. In contrast, for the real ZhengMix datasets, we subsampled and mixed cells of different types using weighted random sampling based on the target size of cell types to ensure diverse cluster sizes and ratios.
Data exclusions	No specific data was excluded.
Replication	To ensure the reproducibility of our signal detection method, we conducted a signal robustness test by performing ten perturbations and estimating the average angle change of each signal vector across these perturbations.
Randomization	We measured the amounts of non-binary information using the ΔSIL scores, which were calculated by randomly shuffling the non-zero values of datasets with known ground truth labels.
Blinding	Our study does not involve group allocation that requires blinding.

Reporting for specific materials, systems and methods

We require information from authors about some types of materials, experimental systems and methods used in many studies. Here, indicate whether each material, system or method listed is relevant to your study. If you are not sure if a list item applies to your research, read the appropriate section before selecting a response.

Materials & experimental systems

n/a	Involved in the study
☒	☐ Antibodies
☒	☐ Eukaryotic cell lines
☒	☐ Palaeontology and archaeology
☒	☐ Animals and other organisms
☒	☐ Clinical data
☒	☐ Dual use research of concern
☒	☐ Plants

Methods

n/a	Involved in the study
☒	☐ ChIP-seq
☒	☐ Flow cytometry
☒	☐ MRI-based neuroimaging

Plants

Seed stocks	*Report on the source of all seed stocks or other plant material used. If applicable, state the seed stock centre and catalogue number. If plant specimens were collected from the field, describe the collection location, date and sampling procedures.*
Novel plant genotypes	*Describe the methods by which all novel plant genotypes were produced. This includes those generated by transgenic approaches, gene editing, chemical/radiation-based mutagenesis and hybridization. For transgenic lines, describe the transformation method, the number of independent lines analyzed and the generation upon which experiments were performed. For gene-edited lines, describe the editor used, the endogenous sequence targeted for editing, the targeting guide RNA sequence (if applicable) and how the editor was applied.*
Authentication	*Describe any authentication procedures for each seed stock used or novel genotype generated. Describe any experiments used to assess the effect of a mutation and, where applicable, how potential secondary effects (e.g. second site T-DNA insertions, mosiacism, off-target gene editing) were examined.*
