# Supplementary material for: scLENS: data-driven signal detection for unbiased scRNA-seq data analysis
Source: Nat Commun. 2024 Apr 27;15:3575. doi: 10.1038/s41467-024-47884-3 (PMC11519519; doi:10.1038/s41467-024-47884-3)
Supplement: Supplementary file 7 — Response to Editorial Requests [file 41467_2024_47884_MOESM7_ESM.docx]

Dear editorial team,

Thank you for your guidance. We have made all necessary revisions to our article, including updates to the DOI, adjustments in document formatting, and clarifications on image generation and source data referencing. Please find attached a detailed response for each point addressed.

**ARTICLE FILE:**

1) Please provide the DOI within the code availability statement

We have now added DOI within the code availability statement as follows:

• Page 27 (code availability statement)

**Code availability**

The Julia codes for the scLENS, and the codes for the R and Python packages used in the benchmark study are available in the Mathbio GitHub:  <https://github.com/Mathbiomed/scLENS>. The package version used for the analyses in the paper has been assigned a citable DOI through Zenodo *(*[*https://doi.org/10.5281/zenodo.10839592*](https://doi.org/10.5281/zenodo.10839592)*)*^76^.

2) Please remove the supplementary information section from the end of your article file, this is not needed.

Following this request, the supplementary information section has been removed from the end of the article file.

SUPPLEMENTARY INFORMATION:

3) Please upload the supplementary information file as a .pdf

The supplementary information file has been converted to a PDF format and uploaded accordingly.

**FIGURES:**

4) Please provide more information, in your response here, on the software used to generate the images in 1.

Or, please provide evidence of the permissions you have obtained in order to commercially reproduce these images

We created Figure 1 using Adobe Illustrator.

5) Please mention the source data files in all relevant figure and supplementary figure legends using the template text below: 'Source data are provided as a Source Data file.' A reference to the source data file should be added at the end of the 'Data Availability' section, using the text “Source data are provided with this paper.”

We have updated all relevant figure and supplementary figure legends, as well as the 'Data Availability' section, to include the appropriate phrase: "Source data are provided with this paper" or "Source data are provided as a Source Data file."

• Page 27 (data availability)

The mouse embryos dataset^55^ was obtained from the Hemberg Lab repository [[https://hemberg-lab.github.io/scRNA.seq.datasets/mouse/edev/#goolam](https://hemberg-lab.github.io/scRNA.seq.datasets/mouse/edev/%23goolam)]. The colorectal tumor cells dataset^56^ was obtained from the Hemberg Lab repository [[https://hemberg-lab.github.io/scRNA.seq.datasets/human/tissues/#li](https://hemberg-lab.github.io/scRNA.seq.datasets/human/tissues/%23li)]. *Source data for Figs. 4-7 and Supplementary Figs. 6-7 have been provided with this paper.* A selection of the real datasets and all simulated datasets used in this study are available in the GitHub repository [<https://github.com/Mathbiomed/scLENS>] and archived at Zenodo^76^.

**RESPONSE TO EDITORIAL REQUESTS:**

6) Please provide a point-by-point response letter in response to the new round of editorial requests.

A point-by-point response letter has been prepared and submitted, addressing each of the new editorial requests.
